# Acute tubulointerstitial nephritis complicating Legionnaires' disease: a case report

**DOI:** 10.1186/1752-1947-6-100

**Published:** 2012-04-04

**Authors:** Aurélie Daumas, Fadwa El-Mekaoui, Stanislas Bataille, Laurent Daniel, Jean-Marie Caporossi, Pierre-Edouard Fournier, Stéphane Burtey, Bertrand Dussol, Yvon Berland, Noémie Jourde-Chiche

**Affiliations:** 1 Service de Néphrologie, Dialyse et Transplantation rénale, Assistance Publique des Hôpitaux de Marseille (AP-HM), Hôpital de la Conception, Bd Baille, F-13005 Marseille, France; 2 Service d'anatomopathologie, Assistance Publique des Hôpitaux de Marseille (AP-HM), Hôpital de la Timone, 264 rue Saint Pierre, F-13005 Marseille, France; 3 Service de Radiologie, Assistance Publique des Hôpitaux de Marseille (AP-HM), Hôpital de la Conception, Bd Baille, F-13005 Marseille, France; 4 Fédération de Microbiologie Clinique, Assistance Publique des Hôpitaux de Marseille (AP-HM), Hôpital de la Timone, 264 rue Saint Pierre, Marseille, and Unité des Rickettsies, Faculté de Médecine, CNRS-IRD UMR6020, F-13005 Marseille, France

**Keywords:** Legionnaires' disease, acute renal failure, tubulointerstitial nephritis, renal biopsy

## Abstract

**Introduction:**

Legionnaires' disease is recognized as a multi-systemic illness. Afflicted patients may have pulmonary, renal, gastrointestinal tract and central nervous system complications. However, renal insufficiency is uncommon. The spectrum of renal involvement may range from a mild and transient elevation of serum creatinine levels to anuric renal failure requiring dialysis and may be linked to several causes. In our present case report, we would like to draw attention to the importance of the pathological documentation of acute renal failure by reporting a case of a patient with acute tubulointerstitial nephritis complicating Legionnaires' disease.

**Case presentation:**

A 55-year-old Caucasian man was admitted to our hospital for community-acquired pneumonia complicated by acute renal failure. *Legionella pneumophila *serogroup type 1 was diagnosed. Although the patient's respiratory illness responded to intravenous erythromycin and ofloxacin therapy, his renal failure worsened, he became anuric, and hemodialysis was started. A renal biopsy was performed, which revealed severe tubulointerstitial nephritis. After initiation of steroid therapy, his renal function improved dramatically.

**Conclusions:**

This case highlights the importance of kidney biopsies in cases where acute renal failure is a complicating factor in Legionnaires' disease. If the presence of acute tubulointerstitial nephritis can be confirmed, it will likely respond favorably to steroidal treatment and thus irreversible renal damage and chronic renal failure will be avoided.

## Introduction

Legionnaires' disease (LD), caused by the bacterium *Legionella pneumophila*, is a leading cause of severe community-acquired pneumonia. It is associated with frequent extrapulmonary symptoms. Acute tubulointerstitial nephritis (TIN) is a rare complication of LD. We report the case of a 55-year-old Caucasian man with anuric acute renal failure (ARF) in a context of LD. A renal biopsy showed severe acute TIN which responded remarkably well to steroid therapy. These findings suggest that when ARF develops in a patient with LD, TIN should be considered as one of the differential diagnoses. Furthermore, this case highlights the importance of renal histology in cases of ARF in LD, because, if acute TIN is documented, systemic corticosteroid therapy may be an effective treatment of ARF, and its rapid initiation may spare the patient from future renal scarring and chronic renal failure.

## Case presentation

A 55-year-old Caucasian man was admitted to the Nephrology Department at our institution for ARF diagnosed in the emergency room along with left-sided, community-acquired pneumonia. He was on oral anti-diabetic treatment for uncomplicated type 2 diabetes and was a cigarette smoker. He reported no recent use of non-steroidal anti-inflammatory drugs or antibiotics.

Clinical examination revealed that his temperature was 38°C and his blood pressure was 120/60 mmHg. His urinary output was diminished and concentrated. Pulmonary examination revealed diffuse crackles of the left lung accompanied by a dry, irritative cough and exertional dyspnea. The rest of the patient's examination was normal.

Chest X-ray revealed alveolar opacities in the left lung. No sputum could be obtained for culture, but his test for *Legionella *antigenuria was positive. Antibiotic therapy with erythromycin and ofloxacin was initiated.

Blood tests revealed elevated serum creatinine (614 μmol/L; normal range, 62 to 106 μmol/L), blood urea nitrogen (28 mmol/L; normal range, 2.14 to 7.14 mmol/L) and C-reactive protein (360 mg/L; normal range, 0 to 3 mg/L) with leukocytosis (13 g/L; normal range, 4 to 11 g/L). No anemia or thrombocytopenia was noted, and the patient's liver function tests were normal. The patient had elevated levels of lactate dehydrogenase (408 IU/L; normal range, 135 to 225 IU/L) and creatine phosphokinase (CPK) (2000 IU/L; normal range, 47 to 171 IU/L). At room air, his arterial blood gas was pH 7.44 (normal range, 7.35 to 7.45), partial pressure of carbon dioxide was 29 mmHg (normal range, 35 to 45 mmHg) and partial pressure of oxygen was 65 mmHg (normal range, 80 to 100 mmHg) with HCO_3 _of 22 mmol/L (normal range, 20 to 25 mmol/L).

Analysis of the urinary sediment revealed aseptic leukocyturia (684/mm^3^; normal range, < 20/mm^3^) and hematuria (56/mm^3^; normal range, 0 to 10/mm^3^). The patient's urinary sodium was below 20 mmol/L, urinary urea was 13 g/L and proteinuria was 2.48 g/L (normal range, 0 to 0.3 g/L) with albuminuria of 0.4 g/L (normal range, < 0.03 g/L). His renal ultrasound was normal.

Although our patient's respiratory signs and chest X-ray revealed improvement with antibiotics, his ARF worsened despite saline solute infusion, and he became anuric. His serum creatinine level at day 3 was 1000 μmol/L. Hemodialysis was initiated with a central jugular catheter.

**Figure 1:**
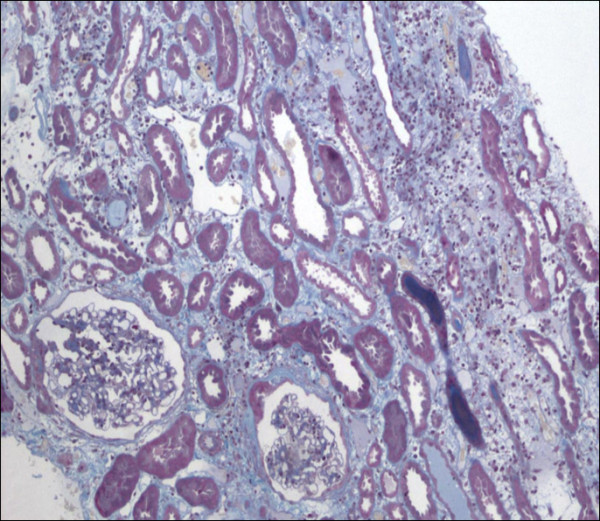
**Renal biopsy**. Renal biopsy showing interstitial cell infiltrate associated with edema and few tubules lined by flattened cells. No granuloma was observed. Masson trichrome stain; original magnification, ×100.

**Figure 2:**
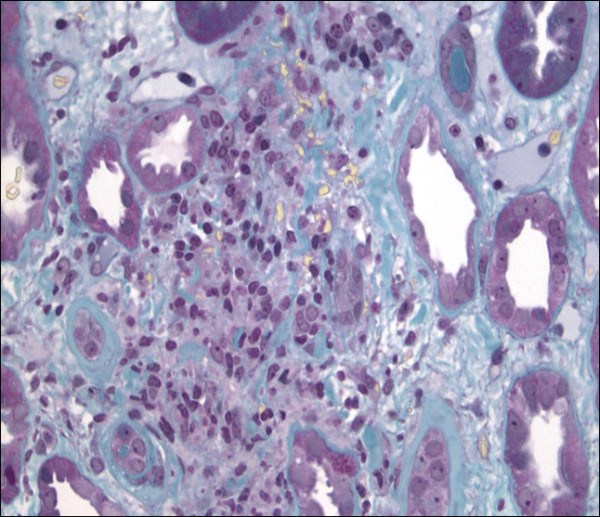
**Renal biopsy**. Renal biopsy showing focal tubulitis with mononuclear cells that have invaded few tubules. No granuloma was observed. Masson trichrome stain; original magnification, ×200.

Percutaneous renal biopsy was performed at day 4, which showed acute TIN (Figures 1 and 2) with interstitial edema and inflammatory peritubular infiltrate composed of lymphocytes and plasma cells. No proliferation or deposit was noted on the 21 glomeruli examined. Under immunofluorescence, only immunoglobulin-secreting plasma cells were visible.

The patient's blood cultures were normal, his bacterial and viral serologies were negative (leptosirosis, human immunodeficiency virus (HIV), hepatitis B virus and hepatitis C virus), the search for tuberculosis and autoimmunity was negative (normal complement level, negative anti-nuclear antibodies and anti-SSA/SSB) and his eye examination was normal. Therefore, we attributed his acute TIN to LD.

Steroid treatment was initiated at 1 mg/kg/day. The patient's renal function rapidly improved, with appropriate diuresis allowing for withdrawal of hemodialysis after 2 days. There was no worsening of respiratory signs under steroid treatment. The patient was discharged at day 10, at which time his serum creatinine level was 110 μmol/L. One month later, after cessation of steroids and antibiotics, his serum creatinine level was 77 μmol/L. Investigation by Health Services did not find the source of *Legionella *contamination.

## Discussion

LD was named after an epidemic that erupted in 1976 among 182 participants in the 58th Congress of the American Legion in Philadelphia. *Legionella *are Gram-negative coccobacilli with several serogroups. *L. pneumophila *is most often involved (90% to 98%), especially serogroup 1, which is responsible for 67% to 90% of all cases of LD [1]. Legionellosis can present as two distinct clinical entities: LD, pneumonia with multi-systemic disease, and Pontiac fever, a non-pneumonic flu-like disease [1].

LD is transmitted from the environment to humans by inhalation of an infectious aerosol. The risk factors are male sex, advanced age, nicotine addiction, alcoholism, diabetes mellitus, respiratory and cardiovascular diseases, immunodepression (malignancies and immunosuppressive treatments) and ventilation and home aerosols [1]. Contamination of collective water networks (in hospitals, hotels, campsites and spa resorts, for example) or water-cooling towers is also a potential source of infection and must be considered in all cases of LD [1]. Declaration of the disease is thus compulsory.

LD is one of the three most common causes of severe community-acquired acute pneumonia in Europe and in up to 40% of cases of hospital-acquired pneumonia [1]. There is no radiological or clinical specificity of LD pneumonia. Nevertheless, some features are particularly evocative: a nosocomial or epidemic context; a very suggestive clinical tableau (one-third of cases) comprising severe pneumonia, acute onset, absence of ear, nose and throat symptoms, pulse dissociated from body temperature, bilateral involvement, abdominal signs and neurological signs; early biological signs comprising hepatic cytolysis, renal failure, hyponatremia, hypophosphatemia and increased CPK; failure of a previous β-lactam antibiotic therapy; and an immunocompromised patient.

The urine antigen test is highly specific, provides rapid results and is particularly useful, because positive *Legionella *antigenuria can persist for days, even during administration of antibiotics. Yet, it detects only *L. pneumophila *serogroup 1 [1-3], and a negative antigen test does not exclude legionellosis with 100% accuracy [4]. The risk of false-positive results has been reported in patients receiving anti-thymocyte drugs and in those with rheumatoid-like factors in urine [4]. Sputum cultures have a high sensitivity and specificity and allow for the identification of all types of *Legionella*; however, obtaining an adequate sputum specimen can be difficult, as was the case in our patient [1-5]. The test for serum antibodies to *Legionella *has a high specificity but the lowest sensitivity, with a fourfold increase in antibody titers being necessary for the assessment of seroconversion, which may not be detectable until 4 to 12 weeks after infection [3].

To date, clinical experience has not shown polymerase chain reaction (PCR) to be more sensitive than cultures, and therefore the US Centers for Disease Control and Prevention does not recommend the routine use of genetic probes or PCR for the detection of *Legionella *in clinical samples [2]. According to the guidelines for the management of adult lower respiratory tract infections [2], efforts should be made to detect urinary *L. pneumophila *serogroup 1 antigen in patients admitted to the hospital for reasons of severity and in other patients in whom the infection is clinically or epidemiologically suspected, but specific culture is always indicated [4]. The availability of the rapid urine test for *Legionella *antigen has decreased the time to diagnosis [1,2].

Current therapeutic recommendations [1,2] propose the use of macrolide or fluoroquinolone monotherapy in cases involving the common forms of LD in immunocompetent patients. In severe forms of LD, or in an immunocompromised patient, the association of two intravenous antibiotics from among the following three is recommended: macrolide, fluoroquinolone and rifampicin. The duration of treatment is classically 14 to 21 days for an immunocompetent subject and can be extended to 30 days in immunocompromised patients or in those with severe forms of LD.

LD is recognized as a multi-systemic illness [1,3]. Patients may have pulmonary, gastrointestinal tract and central nervous system complications. Even if microscopic hematuria is frequently encountered [6], ARF is an uncommon finding in LD.

The mechanism of renal failure associated with LD is mostly multi-factorial, and, in addition to functional ARF (hypovolemia), acute tubular necrosis (shock or rhabdomyolysis) and drug toxicity, *L. pneumophila *also has its own renal toxicity [7-14]. The mechanism of renal dysfunction could be a direct nephrotoxicity of the microorganism, but the presence of *Legionella *bacteria in renal tissue has been documented by electron microscopy in only three cases [8]. In the lung, the organism is phagocytosed into respiratory epithelial cells, where it replicates and induces cellular injury. The same process may occur in renal epithelial cells [9]. In our observation, bacterial antigens were not found in renal tissue. The most likely explanation for the systemic manifestations of the LD, including ARF, is the presence of a circulating endotoxin responsible for vasoconstriction or occlusion of the microvasculature of various organs [10].

Histological examination of renal biopsies in patients with ARF in the context of LD usually shows TIN and/or acute tubular necrosis [7-14]. In 1978, Relman and McCluskey described a case of acute TIN in a patient with pulmonary LD [15] followed in 1981 by reports by Poulter *et al. *[7] and Carlier *et al. *[11]. In 1987, Haines *et al. *[12] described for the first time the inaugural renal involvement of legionellosis without previous respiratory involvement. More recently, Verhaeverbeke *et al. *[13] reported acute TIN during LD with a favorable outcome without administration of corticosteroids after antibiotics and temporary hemodialysis.

An interesting review by Nishitarumizu *et al. *[14] illustrates different causes of ARF in LD. They reported 45 cases of ARF in a context of LD, among whom 15 had a renal biopsy showing the following results: TIN in 5, acute tubular necrosis in 6, crescentic glomerulonephritis in 1, proliferative mesangial glomerulonephritis in 1 and pyelonephritis in 2. Hemodialysis was necessary in 55.5% of these cases, and the mortality rate reached 51% (versus 15% in patients without ARF).

**Figure 3:**
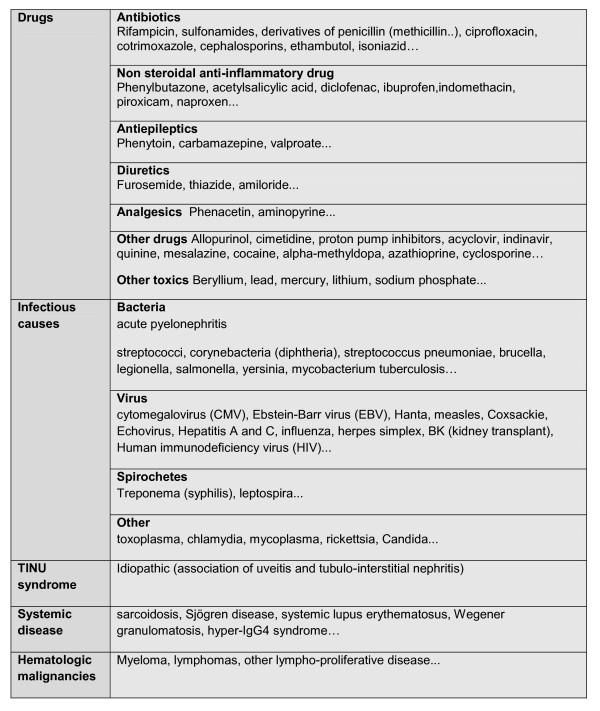
**Causes of acute tubulointerstitial nephritis**.

There is no biological or pathological specificity of TIN associated with LD. The diagnosis is made based upon the clinical context and elimination of other causes of acute TIN, especially drug-induced TIN (Figure 3).

Patients with acute TIN present with ARF, sometimes oligoanuric. The presence of tubular proteinuria (positive proteinuria with no or few albuminuria), aseptic leukocyturia and absence of high blood pressure are suggestive of this diagnosis. The presence of rash, fever or hypereosinophilia is suggestive but inconstant.

Renal pathology shows localized or diffuse lymphoplasmacytic infiltrate with interstitial edema and tubular lesions. Few eosinophils may be seen. In the case of LD, *Legionella *antigen can be found by PCR in renal tissue but is inconstant. Non-caseous granuloma is sometimes encountered in drug-induced TIN or in TIN due to tuberculosis, sarcoidosis or TIN and uveitis (the TINU syndrome) but is uncommon in TIN associated with LD. The presence of scarring lesions such as tubular atrophy or interstitial fibrosis worsens the renal prognosis.

The overall mortality rate for LD is reported to be approximately 15% [8]. Delayed treatment or missed diagnosis may lead to higher mortality, and cases complicated by ARF are reported to have increased mortality (53% in the literature review presented by Shah *et al. *[8]).

Because acute TIN due to LD is a rare disease, no controlled clinical study has ever been conducted concerning the use of steroids to improve the renal prognosis. Yet, even if a complete recovery of renal function is possible without steroids [13], the severity of ARF in our observation led us to begin steroid therapy to rapidly decrease renal inflammation [14] and avoid further renal scarring and chronic renal failure.

This case highlights the importance of the renal biopsy in the differential diagnosis of ARF in LD. Assuming that ARF is due to acute tubular necrosis may prevent or delay the initiation of steroid treatment and, as a result, the opportunity to avoid scarring lesions and chronic renal failure.

## Conclusion

We present a new case report of acute TIN associated with LD that was responsible for anuric ARF necessitating hemodialysis, with rapid improvement of renal function when treated with antibiotics and steroids. We would like to draw attention to the importance of the pathological documentation of ARF in the context of LD for the diagnosis of acute TIN that is likely to respond favorably to steroid treatment.

## Consent

Written informed consent was obtained from the patient for publication of this case report and any accompanying images. A copy of the written consent is available for review by the Editor-in-Chief of this journal.

## Competing interests

The authors declare that they have no competing interests.

## Authors' contributions

AD and NJC drafted the manuscript. AD, FEM, StaB and NJC analyzed and interpreted the patient data regarding the infectious disease and the ARF. LD performed the histological examination of the kidney. PEF performed PCR in the renal tissue. JMC lended his expertise on imaging of the patient and helped to draft the manuscript. BD, StéB and YB contributed to the writing of the manuscript. All authors read and approved the final manuscript.
